# Chidamide and venetoclax synergistically exert cytotoxicity on multiple myeloma by upregulating BIM expression

**DOI:** 10.1186/s13148-022-01306-7

**Published:** 2022-07-07

**Authors:** Liqin Cao, Qingxiao Chen, Huiyao Gu, Yi Li, Wen Cao, Yang Liu, Jianwei Qu, Yifan Hou, Jing Chen, Enfan Zhang, Jingsong He, Zhen Cai

**Affiliations:** 1 Bone Marrow Transplantation Center, The First Affiliated Hospital, Zhejiang University School of Medicine, No. 79 Qingchun Rd, Hangzhou, 310003 Zhejiang China; 2 Institute of Hematology, Zhejiang University, Hangzhou, China; 3 Zhejiang Laboratory for Systems and Precision Medicine, Zhejiang University Medical Center, 1369 West Wenyi Road, Hangzhou, China

**Keywords:** HDAC inhibitor, Multiple myeloma, Chidamide, Venetoclax, Cell cycle arrest, DNA damage, BIM

## Abstract

**Background:**

Multiple myeloma (MM) is the second most common hematologic malignancy with almost all patients eventually having relapse or refractory MM (RRMM), thus novel drugs or combination therapies are needed for improved prognosis. Chidamide and venetoclax, which target histone deacetylase and BCL2, respectively, are two promising agents for the treatment of RRMM.

**Results:**

Herein, we found that chidamide and venetoclax synergistically exert an anti-myeloma effect in vitro in human myeloma cell lines (HMCLs) with a combination index (CI) < 1. Moreover, the synergistic anti-myeloma effect of these two drugs was demonstrated in primary MM cells and MM xenograft mice. Mechanistically, co-exposure to chidamide and venetoclax led to cell cycle arrest at G0/G1 and a sharp increase in DNA double-strand breaks. In addition, the combination of chidamide and venetoclax resulted in BCL-X_L_ downregulation and BIM upregulation, and the latter protein was proved to play a critical role in sensitizing HMCLs to co-treatment.

**Conclusion:**

In conclusion, these results proved the high therapeutic potential of venetoclax and chidamide combination in curing MM, representing a potent and alternative salvage therapy for the treatment of RRMM.

**Supplementary Information:**

The online version contains supplementary material available at 10.1186/s13148-022-01306-7.

## Introduction

Multiple myeloma (MM) is a malignant proliferative disease of plasma cells. Although the prognosis of MM has greatly improved with the application of proteasome inhibitors, immunomodulators and immune-targeted therapy, virtually all patients with MM eventually relapse, prompting the need for novel drugs and combination therapies to improve the further prognosis of patients with relapsed or refractory MM (RRMM).

Venetoclax, a ‘BH3-mimetic’ antagonist of the BCL2 anti-apoptotic protein, is currently widely used in some hematological malignancies including chronic lymphocytic leukemia (CLL), acute myeloid leukemia (AML) and mantle cell lymphoma (MCL) [1–6]. In myeloma, sensitivity to venetoclax in vitro is mainly observed in plasma cells harboring the t (11;14) translocation, a molecular subgroup associated with high BCL2 and low MCL1/BCL-X_L_ expression [7]. Phase I clinical trials confirmed the efficacy of venetoclax monotherapy in RRMM patients, mostly in those with t (11;14) translocation [8], followed by a phase III study that confirmed the benefit of venetoclax in combination with bortezomib and dexamethasone when used in patients with RRMM and confined to t (11;14) and high BCL2 expression. In the absence of these biomarkers, venetoclax in combination therapy was associated with higher mortality and an unfavourable risk–benefit profile, despite longer progression-free survival [9]. Mechanistically, venetoclax induces myeloma cell apoptosis by displacing proapoptotic BH3-only proteins (e.g., BIM and PUMA) from BCL2, leading to caspase-dependent cell death [10, 11].

Chidamide, a histone deacetylase inhibitor (HDACi), mainly targets histone deacetylases (HDACs) 1, 2, 3 and HDAC10. HDACis (such as chidamide) were reported to be effective in killing of a variety of tumor cells, especially in hematological malignancies, including MM through inhibiting cell proliferation and inducing cell cycle arrest, DNA damage, autophagy, ferroptosis, and apoptosis [12–17]. It has been reported [18–20] that HDACi (including chidamide) could lead to the dysregulation of anti- and pro-apoptotic BCL2 family proteins, such as upregulating pro-apoptotic proteins (e.g., BAX and BAK) and BH3-only proteins (e.g., BIM, BID and PUMA) and decreasing the levels of pro-survival proteins (e.g., BCL2, BCL-X_L_ and MCL1). Previous study has proved that ventoclax can release BIM from pro-survival proteins and thus promotes BIM to dimer with BAX and BAK, which can induce endogenous apoptosis [21]. In addition, high MCL1/BCL2 or BCL-X_L_/BCL2 mRNA ratio indicates that myeloma cells resist venetoclax [22]. This has raised the possibility that HDACi may be able to interact with venetoclax by the dysregulation of pro-survival and pro-apoptotic BCL2 family proteins to exert cytotoxicity in myeloma cells.

The present study aims to examine the potential synergistic anti-myeloma effect of the combination of chidamide and venetoclax. It was observed that these two agents synergistically exert an anti-myeloma effect in various human myeloma cell lines (HMCLs), primary MM samples and MM xenograft NOD/SCID mice. Mechanistically, the combination of chidamide and venetoclax induced DNA double-strand break, cell cycle arrest, downregulation of BCL-X_L_ and upregulation of BIM, which was demonstrated as essential for the sensitization of HMCLs to the co-treatment.

## Results

### Chidamide and venetoclax demonstrate synergistic anti-myeloma effect in HMCLs in vitro

**Fig. 1:**
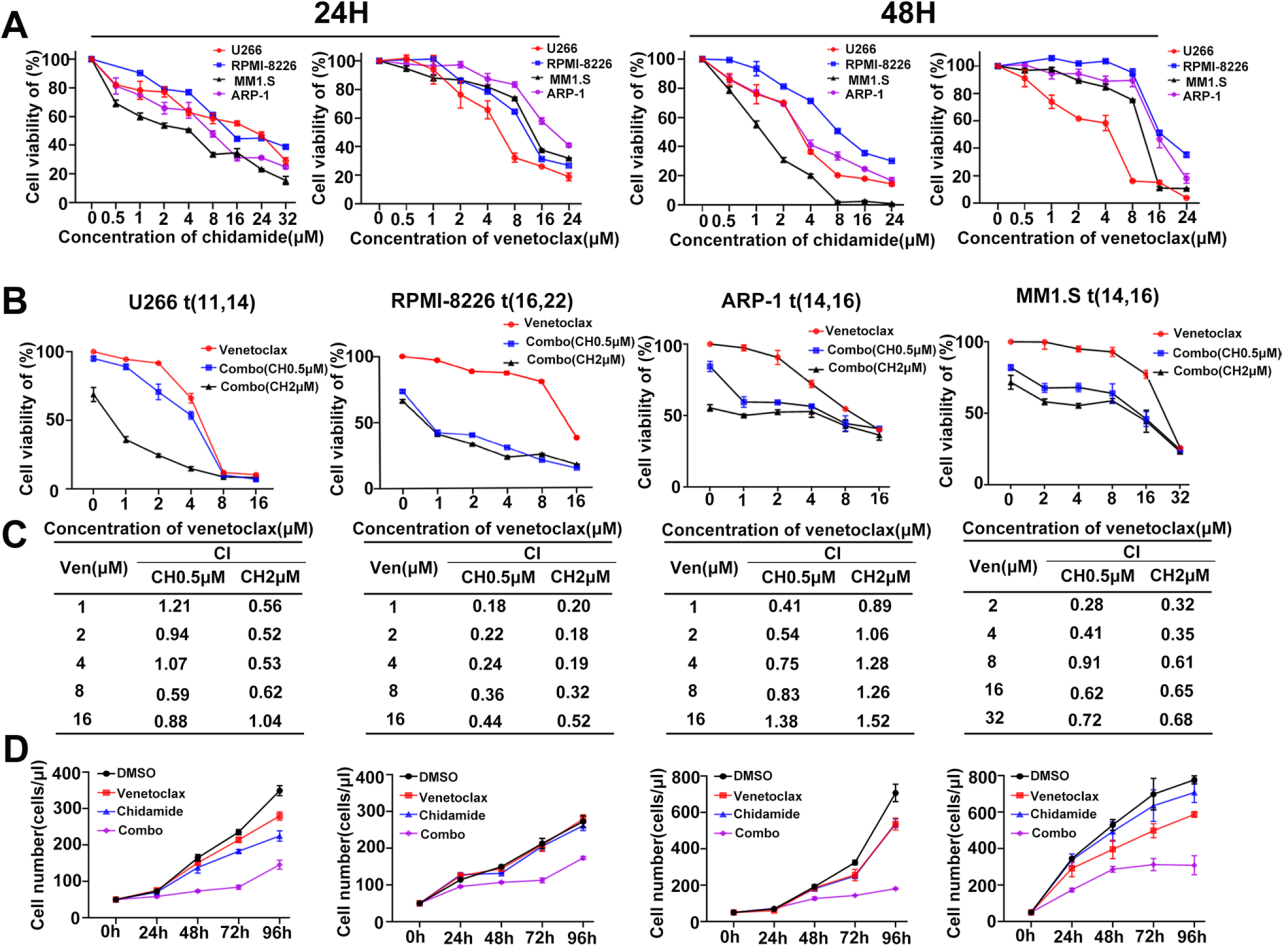
Chidamide and venetoclax demonstrate synergistic anti-myeloma efficacy in HMCLs in vitro. **A** HMCLs were treated with the indicated concentrations of chidamide and venetoclax for 24 h and 48 h, then CCK-8 assay was performed to test cell viability. **B** HMCLs were treated with the indicated concentrations of venetoclax ± 0.5 or 2 μM chidamide for 48 h, and the CCK-8 kit was used to test the cell viability. **C** From the dose–response curves, the CI of the two drugs were calculated using CompuSyn software, with CI < 1 indicating a synergistic interaction. **D** Cell growth was monitored every 24 h for a consecutive 4 days. The values indicate mean ± SD for at least three independent experiments performed in triplicate

We first investigated the potential inhibition of cell growth by chidamide and venetoclax in MM cells. HMCLs (U266, ARP-1, RPMI-8226 and MM1.S) were respectively treated with increasing concentrations of chidamide and venetoclax for 24 h and 48 h. When used separately, chidamide and venetoclax both decreased the myeloma cell viability in a dose- and time-dependent manner (Fig. 1A). Notably, U266 was the most sensitive to venetoclax, which might be due to U266 harboring t (11, 14), thus featuring the highest BCL2 mRNA expression and highest BCL2/MCL1 mRNA ratio compared with the other three MM cells (Additional file 1: Figure S1A, B). Then, we selected two concentrations of chidamide (0.5 μM and 2 μM) combined with a series of concentrations of venentoclax treating HMCLs for 48 h to examine the potential synergistic anti-myeloma effect. As shown in Fig. 1B, co-exposure to chidamide and venetoclax resulted in sharply reduced cell viability in all selective cell lines, and the synergy of the two drugs was observed for various venetoclax doses in all cell lines with CI < 1 (Fig. 1C). Moreover, to investigate if the synergistic anti-myeloma effect was sustained over time, cell growth curves were examined every 24 h for consecutive 4 days. As shown in Fig. 1D, the combined treatment showed a more obvious suppressive effect on the growth of HMCLs than chidamide or venetoclax administered individually.

**Fig. 2:**
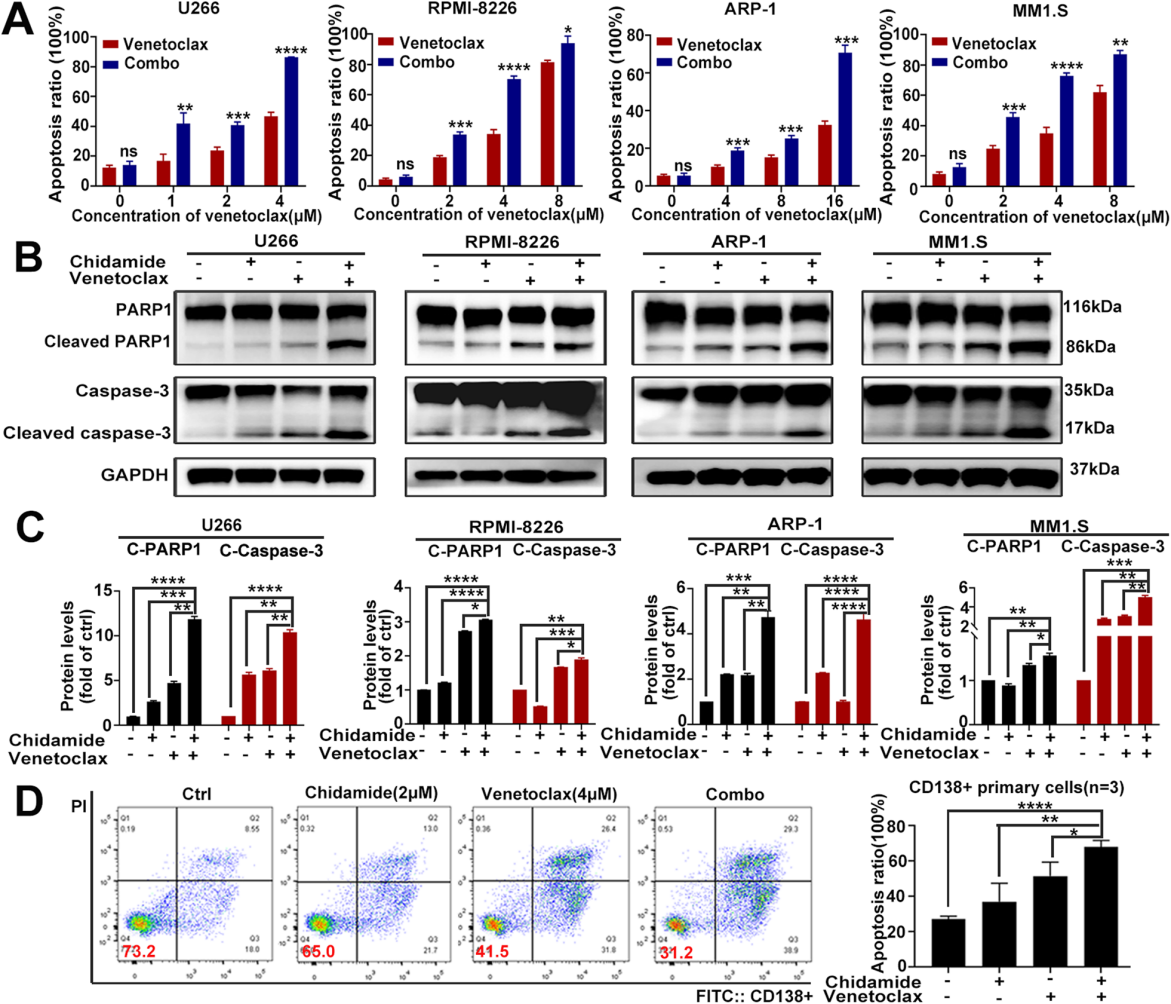
Chidamide and venetoclax demonstrate synergistic anti-myeloma efficacy in HMCLs in vitro. **A** HMCLs were incubated with chidamide (2ɥM), venetoclax (concentration was indicated in picture), their combination or vehicle for 48 h and subjected to flow cytometry to analyze cell apoptosis. **B** HMCLs were incubated with chidamide (1ɥM for U266; 2ɥM for ARP-1, MM1.S and RPMI-8226), venetoclax (2ɥM for U266; 4ɥM for ARP-1, MM1.S and RPMI-8226), their combination or vehicle for 48 h, and the expression of the following apoptosis-related proteins was determined by western blot analysis: PARP1, caspase-3. **C** Protein levels of cleaved PARP1 and cleaved caspase 3 were normalized to those of GAPDH and presented as fold changes relative to vehicle controls. **D** Primary MM samples (*n* = 3) were exposed to chidamide (2ɥM) and/or venetoclax (4ɥM) for 48 h and analyzed by flow cytometry. Data are presented as the mean ± SD of at least three independent experiments (ns *P* > 0.05; ∗ *P* < 0.05; ∗  ∗ *P* < 0.01; ∗  ∗  ∗ *P* < 0.001; ∗  ∗  ∗  ∗ *P* < 0.0001)

We next examined the synergistic effect of co-treatment on apoptosis induction in HMCLs. As shown in Fig. 2A, treatment with combination therapy markedly increased apoptosis compared to any monotherapies in HMCLs. Moreover, cleaved caspase-3 and cleaved PARP1, two apoptosis-related proteins, were significantly increased by combined treatment (Fig. 2B, C).

To further confirm these findings in primary MM samples, we measured apoptosis in CD138 + plasma cells and treated them with chidamide, venetoclax, their combination, or vehicle for 48 h. Similar to our findings in HMCLs, co-treatment induced a higher level of apoptosis of CD138 + plasma cells from MM patients than chidamide or venetoclax administered alone (Fig. 2D). The clinical characteristics of MM patients were shown in Table 1.

**Table 1:** Patient clinical characteristics

Number	Gender	Age	Disease	Isotype	FISH in diagnosed
Patient#1	Male	40	Newly diagnosed MM	IgG, kappa	17p-, t (4,14)
Patient#2	Male	63	RRMM	IgA, kappa	1q21
Patient#3	Female	73	RRMM	IgG, lamda	14q32, 1q21

Taken together, these data revealed that chidamide and venetoclax could synergistically exert cytotoxic effects in HMCLs and primary MM samples.

### Co-treatment with chidamide and venetoclax induces cell cycle arrest at the G0/G1 phase in HMCLs via activating P21 and P27.

**Fig. 3:**
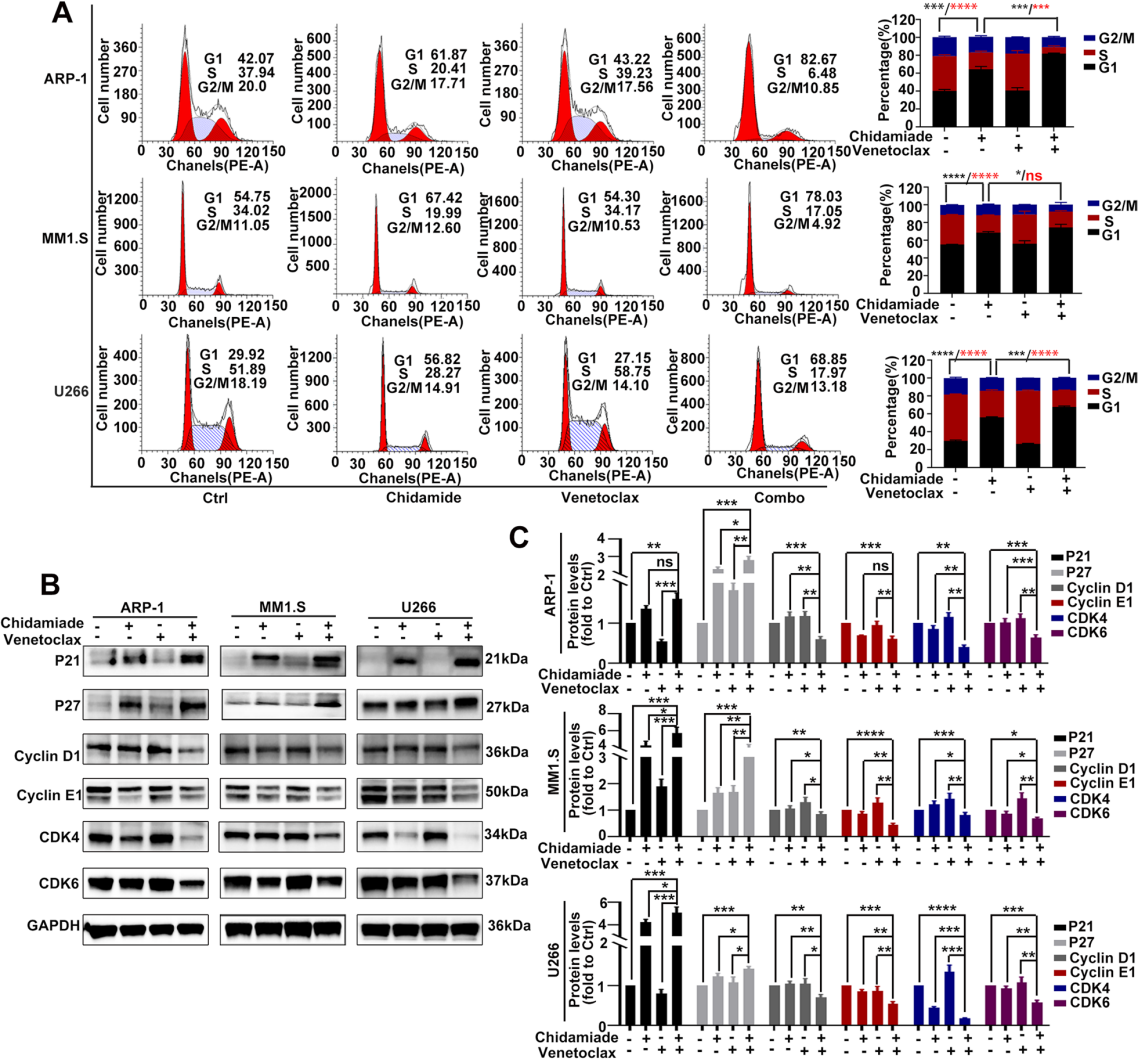
Co-treatment with chidamide and venetoclax induces cell cycle arrest at the G0/G1 phase in HMCLs via activating P21 and P27. **A** HMCLs were incubated with chidamide (1ɥM for U266; 2ɥM for ARP-1 and MM1.S) and/or venetoclax (2ɥM for U266; 4ɥM for ARP-1 and MM1.S) for 48 h, and the cell cycle was analyzed by flow cytometry. **B** HMCLs were exposed to chidamide (1ɥM for U266; 2ɥM for ARP-1 and MM1.S) and/or venetoclax (2ɥM for U266; 4ɥM for ARP-1 and MM1.S) for 48 h. Western blotting was employed to detect the expression of the following cell cycle-related proteins: P21, P27, cyclin D1, cyclin E1, CDK4 and CDK6. **C** Protein levels of P21, P27, cyclin D1, cyclin E1, CDK4 and CDK6 were normalized to those of GAPDH and presented as fold changes relative to vehicle controls. Data are presented as the mean ± SD of at least three independent experiments. (ns *P* > 0.05; ∗ *P* < 0.05; ∗  ∗ *P* < 0.01; ***P < 0.001; ∗  ∗  ∗  ∗ *P* < 0.0001)

In order to further characterize the role of chidamide and venetoclax-mediated cytotoxicity, we evaluated the cell cycle status. In ARP-1, MM1.S and U266 cell lines, the percentage of cells in the S phase was significantly decreased and the ratio of G0/G1 phase was dramatically increased after exposure to chidamide alone for 48 h, while venetoclax alone did not affect the distribution of cell cycle phases (Fig. 3A). Interestingly, co-treatment resulted in a more remarkable cell cycle arrest at the G0/G1 phase compared with chidamide alone in HMCLs (Fig. 3A), which may be associated with the synergy of inducing MM apoptosis. To further explore the molecular mechanisms of cell cycle arrest induced by chidamide or co-treatment, we performed western blotting to assess the expression of cell cycle related proteins. The results showed that the expression of cyclin-dependent kinase inhibitors (CDKIs) P21 and P27, which can block the formation of dimers from cyclins and cyclin dependent kinases (CDKs), were increased in the mono-chidamide group and the co-treatment group. In addition, the cell cyclins, cyclin D1 and cyclin E1 as well as CDKs, CDK4 and CDK6 were remarkably decreased in the co-treatment group (Fig. 3B, C). These findings may suggest that chidamide combined with venetoclax induces cell cycle arrest at the G0/G1 phase in HCMLs by increasing the expression of CDKIs (P21 and P27) and decreasing the expression of cyclins (cyclin D1 and cyclin E1) and CDKs (CDK4 and CDK6).

### Co-treatment with chidamide and ventoclax disrupts DNA damage response and results in DNA damage in MM cells

**Fig. 4:**
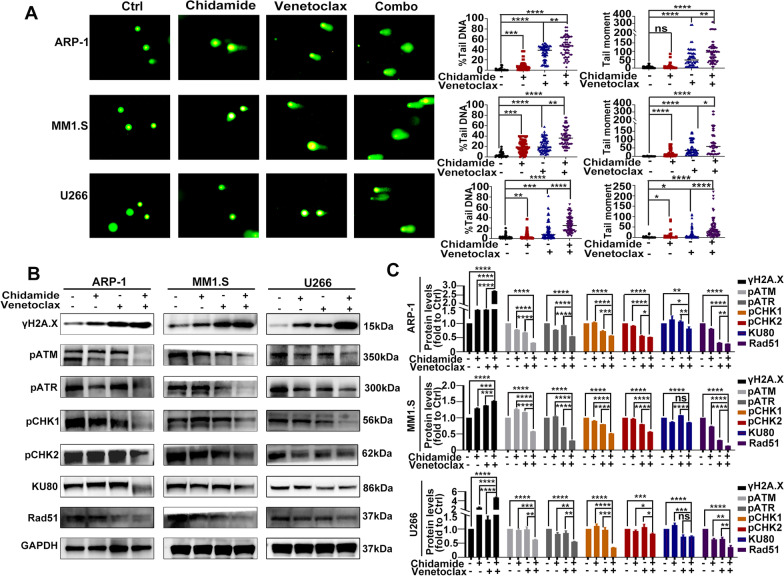
Co-treatment with chidamide and ventoclax disrupts DNA damage response and results in DNA damage in MM cells. **A** HMCLs were incubated with chidamide (1ɥM for U266; 2ɥM for ARP-1 and MM1.S) and/or venetoclax (2ɥM for U266; 4ɥM for ARP-1 and MM1.S) for 48 h, and DNA damage was detected by Comet assay. **B** HMCLs were exposed to chidamide (1ɥM for U266; 2ɥM for ARP-1 and MM1.S) and/or venetoclax (2ɥM for U266; 4ɥM for ARP-1 and MM1.S) for 48 h. Western blotting was used to analyze the expressions of *γ*H2A.X, p-ATM, p-ATR, p-CHK1, p-CHK2, Rad51 and KU80. **C** Protein levels of *γ*H2A.X, p-ATM, p-ATR, p-CHK1, p-CHK2, Rad51 and KU80 were normalized to those of GAPDH and presented as fold changes relative to vehicle controls. Data are presented as the mean ± SD of at least three independent experiments (ns *P* > 0.05; ∗ *P* < 0.05; ∗  ∗ *P* < 0.01; ∗  ∗  ∗ *P* < 0.001; ∗  ∗  ∗  ∗ *P* < 0.0001)

Several studies have shown that chidamide can cause DNA damage in tumor cells [23–25], which can lead to genomic instability and induce endogenous cell apoptosis. We thus examined whether DNA damage would contribute to enhanced cytotoxic effects of the combination of chidamide and venetoclax. First, we used the Comet assay to detect DNA damage. As shown in Fig. 4A, co-treatment with chidamide and venetoclax resulted in higher levels of DNA damage than treatment with vehicle and chidamide or venetoclax alone in HMCLs, manifested by the highest percentages of tail DNA and tail moment. Next, we confirmed these results by western blotting (Fig. 4B, C). As expected, γH2A.X, an established marker for DNA double-strand breaks (DSB) [26], was sharply increased by co-treatment with chidamide and venetoclax in HMCLs. Finally, we explored the mechanism of increasing DNA damage by combination therapy. Generally, DNA damage response (DDR) can identify and repair DNA damage through various pathways and enzymes, and is critical to maintaining genomic stability and cell survival [27]. Notably, combined treatment with these two agents almost completely inhibited the phosphorylation (activation) of DNA damage checkpoints ATM and ATR and thus inhibited the phosphorylation of CHK1 and CHK2, the downstream DNA damage checkpoints of ATM and ATR. Combined treatment also markedly downregulated the expression of DNA repair proteins, Rad51and KU80 (Fig. 4B, C). Altogether, combined treatment induced abundant DNA damage in HMCLs by the disruption of DNA damage checkpoints (e.g., ATM and ATR and their downstream kinases CHK1 and CHK2), as well as by downregulating DNA repair proteins (e.g., Rad51and KU80), which might contribute to the synergistic interaction between chidamide and venetoclax in HMCLs.

### Co-exposure to chidamide and venetoclax induces the apoptosis of HMCLs in connection with BIM upregulation

**Fig. 5:**
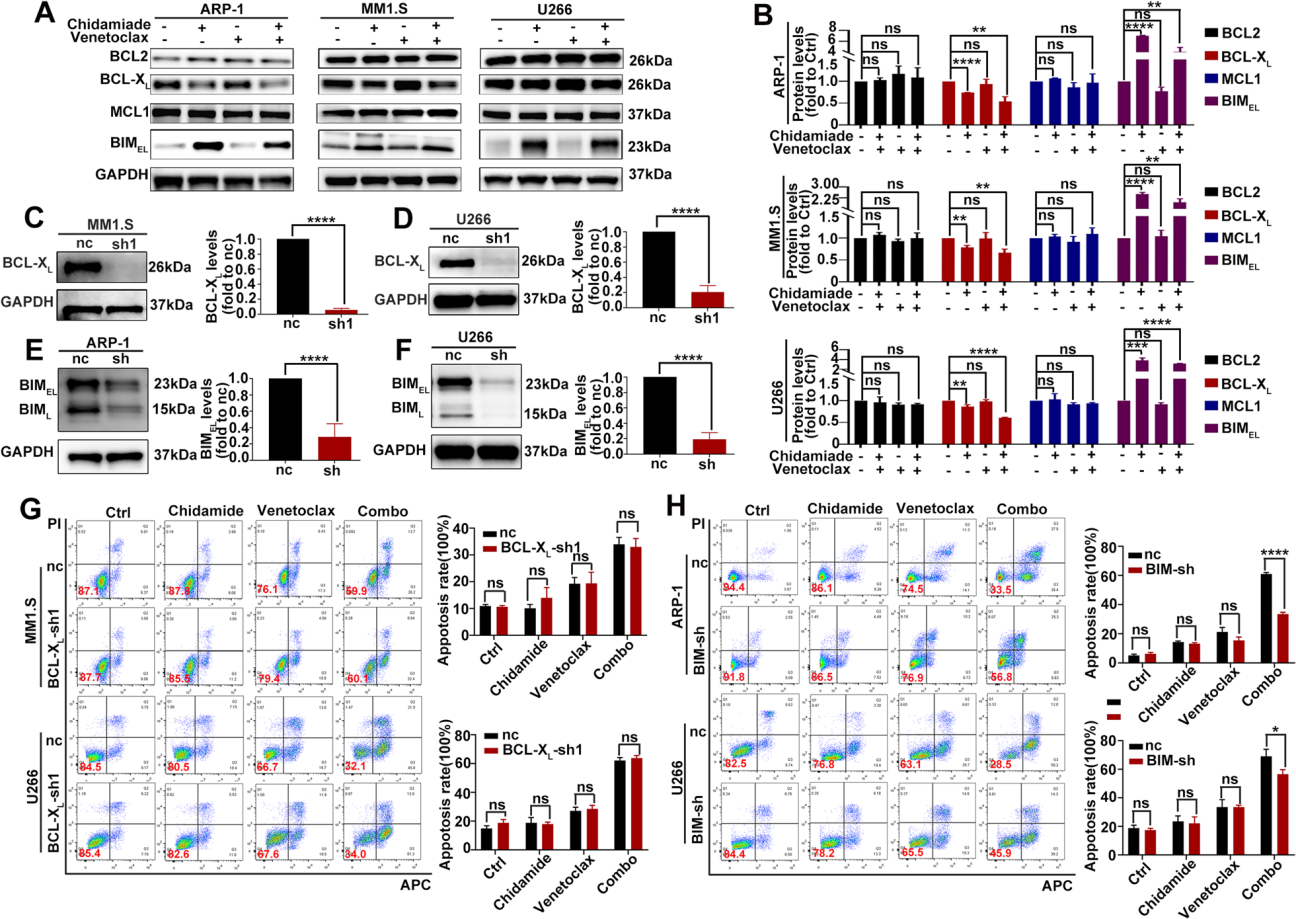
Co-exposure to chidamide and venetoclax induces apoptosis of HMCLs in connection with BIM upregulation. **A** HMCLs were exposed to chidamide (1ɥM for U266; 2ɥM for ARP-1 and MM1.S) and/or venetoclax (2ɥM for U266; 4ɥM for ARP-1 and MM1.S) for 48 h. The expressions of the following BCL2 family proteins were determined by western blot analysis: BCL2, MCL1, BCL-X_L_ and BIM. **B** Protein levels of BCL2, MCL1, BCL-X_L_ and BIM were normalized to those of GAPDH and presented as fold changes relative to vehicle controls. **C** BCL-X_L_ was knocked down by lentivirus in MM1.S cells. **D** BCL-X_L_ was knocked down by lentivirus in U266 cells. **E** BIM was knocked down by lentivirus in ARP-1 cells. **F** BIM was knocked down by lentivirus in U266 cells. **G** MM1.S cells (upper) and U266 cells (down) with BCL-X_L_ knockdown were treated with chidamide (2 μM) ± venetoclax (4 μM) for 48 h and the percentage of apoptosis was determined by flow cytometry **H **ARP-1 cells (upper) and U266 cells (down) with BIM knockdown were treated with chidamide (2 μM) ± venetoclax (4 μM for U266, 8 μM for ARP-1) for 48 h and the percentage of apoptosis was determined by flow cytometry (ns *P* > 0.05; ∗ *P* < 0.05; ∗  ∗ *P* < 0.01; ∗  ∗  ∗ *P* < 0.001; ∗  ∗  ∗  ∗ *P* < 0.0001)

The anti-apoptotic proteins BCL-X_L_ and MCL1 play a critical role in venetoclax resistance, and BH3-only protein BIM is important for venetoclax to exert its cytotoxic effects [28–30]. Moreover, some studies indicated that HDACi can disrupt the expression of anti-apoptotic and pro-apoptotic proteins in the BCL2 family, including decreasing the expression of MCL1 and BCL-X_L_ and increasing the expression of BIM [18–20]. We thus examined the expression change of these BCL2 family proteins after exposure to chidamide and venetoclax. As shown in Fig. 5A, B, the expression of BCL-X_L_ was reduced and BIM was increased in HMCLs after exposure to chidamide in the presence or absence of venetoclax, however, the expression of MCL1 was unchanged by chidamide or venetoclax or their combination. To further confirm which protein was associated with the synergistic interaction between chidamide and venetoclax in HMCLs, we used lentivirus vectors to knock down the BCL2L1 gene (coding BCL-X_L_ protein) in MM1.S and U266 cells (Fig. 5C, D) and knock down the BCL2L11 gene (coding BIM protein) ARP-1 and U266 cells (Fig. 5E, F). The results showed that the downregulation of BCL-X_L_ expression might not account for the synergistic anti-myeloma effect of the two drugs, since combined treatment with chidamide and venetoclax still induced a markedly higher rate of apoptosis than chidamide or venetoclax monotherapy in BCL-X_L_ knockdown cells and the knockdown of BCL-X_L_ did not sensitize MM1.S and U266 cells to chidamide or venetoclax monotherapy or combination therapy (Fig. 5G). In contrast, as shown in Fig. 5H, ARP-1 and U266 cells transfected with shBIM lentiviral vector exhibited lower apoptosis rate than cells transfected with the lentivirus vector when they were treated with the combination regime, while the knockdown of BIM did not protect MM cells from apoptosis induced by venetoclax and chidamide administered individually. In conclusion, these results suggested that the upregulated expression of BIM by chidamide might account for or at least contribute to the synergistic anti-myeloma effect of combined treatment with chidamide and venetoclax.

### Chidamide combined with venetoclax shows synergistic antitumor efficacy in vivo

**Fig. 6:**
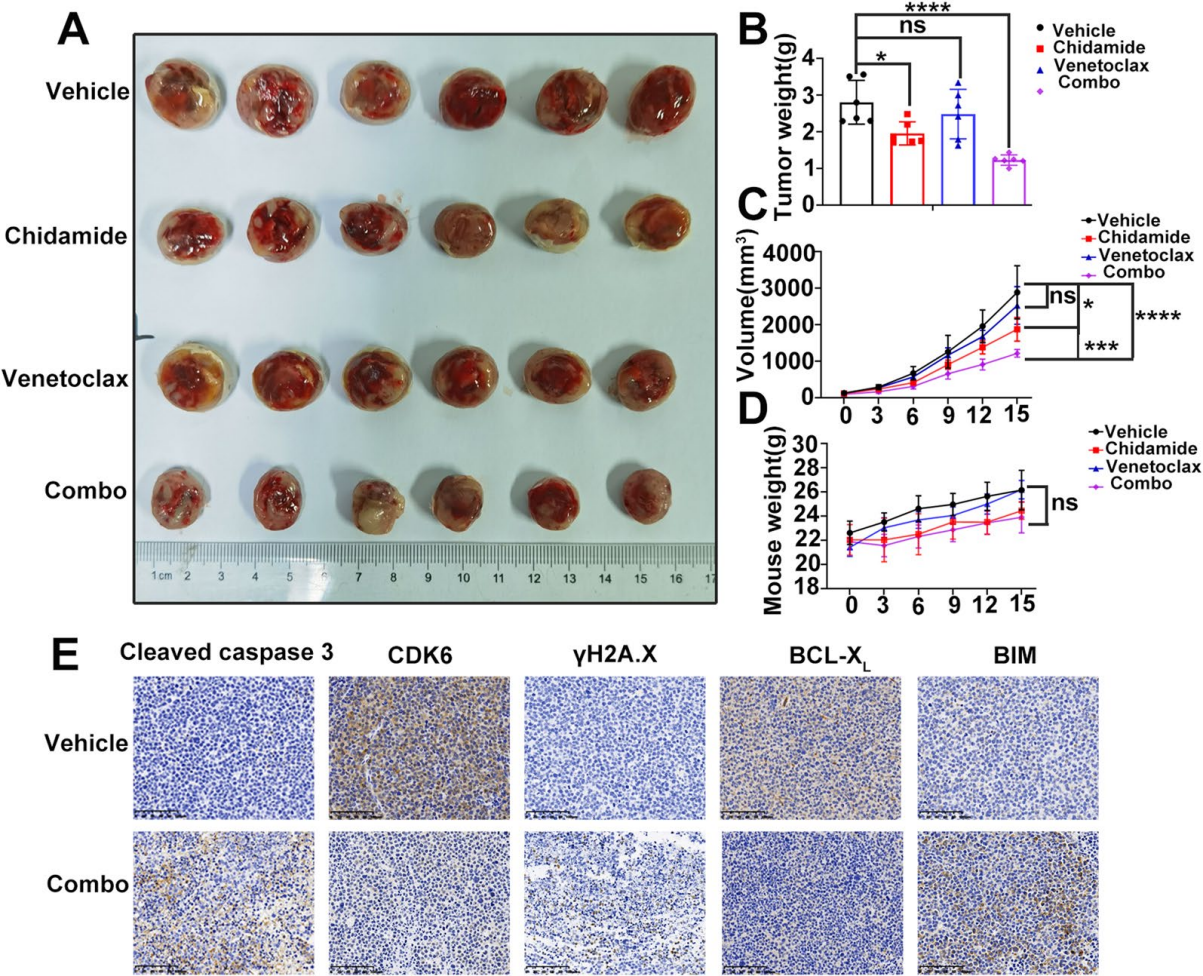
Chidamide in combination with venetoclax shows synergistical antitumor efficacy in vivo. **A** Mice were killed, and their tumor masses were fetched and captured by camera. **B** Tumor weight was measured after detachment. **C** Tumor volumes were measured every three days after tumor formation. **D** Mouse body weight was measured every three days after tumor formation. **E** Immunohistochemistry was performed to investigate the expression of cleaved caspase-3, CDK6, γH2A.X, BCL-X_L_ and BIM in tumor masses. The values indicate mean ± SD for 6 mice/each group (ns *P* > 0.05; *P < 0.05; ***P < 0.001; ****P < 0.0001)

A xenograft mouse model was employed to further validate whether combined treatment with chidamide and venetoclax could synergistically inhibit MM growth in vivo. After 7 days of injecting ARP-1 cells subcutaneously in six-week NOD-SCID mice, the animals were divided into four groups, including vehicle control, chidamide, venetoclax, and the combination of the latter two. Interestingly, as shown in Fig. 6, combined treatment resulted in a more obviously decrease of tumor burden, manifested by reduced tumor volume and tumor weight, compared to vehicle control or chidamide/venetoclax administrated separately (Fig. 6A–C). Moreover, notable toxicity was not observed in mice subjected to combination treatment, as there were no significant weight decreases of mice during the treatment (Fig. 6D).

We next used immunohistochemistry to validate the expression of cleaved caspase-3, CDK6, *γ*H2A.X, BCL-X_L_ and BIM in tumor masses. As expected, combination treatment with chidamide and venetoclax increased the expression of cleaved caspase-3, *γ*H2A.X and BIM, as well as decreased the expression of CDK6 and BCL-X_L_ (Fig. 6E). Collectively, these findings demonstrated that chidamide combined with venetoclax could synergistically inhibit MM growth in vivo.

## Discussion

Although proteasome inhibitors, immunomodulators and immune-targeted therapies have greatly improved the survival of patients with MM, this disease remains incurable, which highlights the urgent need for new agents and drug combinations. Venetoclax, a selective BCL2 antagonist, and chidamide, a novel HDACi, are two promising alternative drugs for the treatment of RRMM. In this study, we proved that chidamide and venetoclax synergistically exert a cytotoxic effect on HMCLs and primary myeloma cells, independently of t (11;14) status. Notably, the synergistic anti-tumor effect was retained in a MM xenograft mouse model.

In myeloma, the sensitivity of cells to venetoclax is strongly associated with the t (11, 14) translocation, since it more likely presents with high BCL2 expression and low MCL1 or BCL-X_L_ expression [10]. In our study, the U266 cell line was more sensitive to venetoclax than the other three cell lines (ARP-1, MM1.S and RPMI 8266), which may be due to U266 harboring the t (11, 14) translocation. Indeed, the qRCR indicated that U266 had higher BCL2 mRNA and BCL2/MCL1 mRNA ratio than the other three cell lines, however, higher BCL2/BCL-X_L_ mRNA ratio were not observed in U266 cells, which may indicate that the sensitivity of MM to venetoclax is associated with high expression of BCL2 and BCL2/MCL1 mRNA ratio but not BCL2/BCL-X_L_ mRNA ratio. Since chidamide mainly targets histone deacetylases class I (HDAC1, HDAC2 and HDAC3) and histone deacetylases class II (HDAC10), we analyzed the expression of HDAC1, HDAC2, HDAC3 and HDAC10 in ARP-1, MM1.S, and U266 cell lines after administrating chidamide and/or venetoclax for 48 h. We found that the expression of HDAC1, HDAC2, HDAC3, and HDAC10 was not affected by monotherapy or combined treatment (Additional file 2: Figure S2A, B), which was consitent with our previous study that indicated that chidamide inhibited HDAC activity and did not affect the expression of HDAC1, HDAC2, HDAC3, and HDAC10 in human MM cell lines [33].

Cell cycle arrest is one of main HDACi-induced cell changes. The main mechanisms of HDACi inducing cell cycle arrest are increased expression of P21 and P27 [31–33], which are CDKIs and can block the formation of dimers from cyclins and CDKs [34, 35]. Moreover, it has been reported that venetoclax could also induce cell cycle arrest at G0/G1 by the inhibition of cell proliferator genes, cyclin D1 and E2F1 [36]. This raises the possibility that these two classes of agents may interact to induce cell cycle arrest at G0/G1 and therefore trigger endogenous cell death. Our findings did not show that venetoclax induced cell cycle arrest, however, chidamide sharply increased the percentage of HMCLs in G0/G1 phase, and more interestingly, the cell cycle arrest induced by chidamide was enhanced by venetoclax. Further analyses showed that co-treatment with chidamide and venetoclax increased the expression of P21 and P27, which combined with cyclins and CDKs (or cyclins-CDKs), and then the blocking of cyclins-CDKs’ activity eventually induced cell cycle arrest [34, 35]. In addition, co-treatment decreased the expression of cyclins (cyclin D1 and cyclin E1) and CDKs (CDK4 and CDK6), which formed complexes and were essential for cells to get through the G1 phase [37, 38]. In conclusion, co-treatment induced cell cycle arrest at the G0/G1 phase though the increased expression of CDKIs and decrease of cyclins and CDKs.

DDR, essential to maintain genomic stability, is often dysregulated in tumor cells, leading to genomic instability. In the case of inadequate or suboptimal DNA repair, the cell accumulates DNA damage and thus activates downstream programmed cell death. HDACs play important roles in the DNA damage response (DDR), which particularly includes DNA damage checkpoint and DNA repair [39]. It has been reported that HDAC inhibitors could induce apoptosis through interference with cytoprotective DDR [40]. In addition, venetoclax can trigger DNA damage by weakening Rad51-meidiated DNA damage repair [41]. Thus, we hypothesized that the DNA damage induced by the combination of the two agents might contribute to the synergistic anti-myeloma effect of the two drugs. Accordingly, our results shown co-exposure to chidamide and venetoclax led to a sharp increase of *γ*H2A.X, a typical marker of DSB. Mechanistically, the co-administration of chidamide and venetoclax inhibited the phosphorylation (activation) of ATM and ATR, two kinases involved in the initiation of DDR [42]. Consequently, the phosphorylation of CHK1 and CHK2, two key DNA damage checkpoint kinases that act as direct downstream targets of ATR and ATM, were also inhibited [43]. Taken together, these results suggest that the combination regimen appears to target ATM and ATR and then inhibit their downstream kinases CHK1 and CHK2. Homologous recombination (HR) repair and non-homologous end joining (NHEJ) repair are two mechanisms of repairing DSB. In our study, the levels of DNA repair proteins Rad51, playing a major role in HR repair, and KU80, essential for non-homologous end joining (NHEJ) repair [44], were decreased by combination treatment, which suggest that the regimen combining chidamide and venetoclax might target DNA repair via both HR and NHEJ repair.

In myeloma, overexpressed MCL1 and BCL-X_L_ leads to myeloma cell survival and resistance to venetoclax therapy [28, 29]. The distribution of the pro-apoptotic protein BIM among BCL2, BCL-X_L_ and MCL1 also plays an important role in the sensitivity of MM cells to venetoclax [30]. Moreover, it was reported that HDACi could inhibit the expression of MCL1 and BCL-X_L_ as well as increase the expression of BIM in a variety of tumor cells [18–20], which may be associated with the synergistical anti-myeloma effect of chidamide and venetoclax. In this study, we found that exposure to chidamide resulted in significant BCL-X_L_ downregulation and BIM upregulation, however, no obvious change in MCL1 expression was indicated. Further research found that the knockdown of BCL-X_L_ did not sensitize MM cells to venetoclax and chidamide administered individually or in combination. These findings support our previous findings that the sensitivity of MM to venetoclax may be not associated with high expression of BCL2/BCL-X_L_. Moreover, the downregulation of BCL-X_L_ by chidamide may not be the main mechanism for the synergistic antitumor effect of the two drugs, as combination treatment still induced remarkably higher percentage of apoptosis than monotherapy in BCL-X_L_ knockdown MM1.S and U266 cells. In contrast, the knockdown of BIM did not protect MM cells from apoptosis induced by venetoclax and chidamide administered individually but made MM cells resist combination therapy. Taken together, these findings suggest that chidamide enhanced the anti-myeloma effect of venetoclax mainly by increasing the expression of BIM.

BCL2 Homology 3 (BH3)-only pro-apoptotic protein, BIM, functions as apoptosis activator as it can directly form oligomers with BAX and BAK and then induce caspase-dependent cell apoptosis [45, 46]. BIM can also bind to pro-survival BCL2 members (BCL2, BCL-X_L_ and MCL1) such that BAX and BAK cannot be activated enough and thus promote cell survival [47, 48]. Venetoclax is a BH3 mimetic that selectively binds to BCL2, release BIM from pro-survival proteins and thereby function as an inhibitor of BCL2 [49]. In this study, chidamide increased the expression of BIM and venetoclax released BIM from BCL2, which resulted in a large amount of free BIM protein exist in cells, forming oligomers with BAX and BAK and inducing caspase-dependent apoptosis. These may be the molecular mechanisms of how the expression of BIM increased through chidamide in MM cells sensitive to venetoclax.

In addition, we found that BIM knockdown could not overcome the increased level of apoptosis induced by combined treatment with chidamide and venetoclax in HMCLs, which implies that other mechanisms such as cell cycle arrest and DNA damage functioned to make MM cells more sensitive to drug combinations.

In summary, the results evidenced the high preclinical efficacy and synergism of combination treatment with chidamide and venetoclax on MM cells, mediated at least in part by the increased expression of BIM. Moreover, cell cycle arrest and DNA damage might also contribute to the synergistic anti-MM effect of the combination treatment. These preclinical findings suggest that chidamide in combination with venetoclax might be an alternative treatment for RRMM in clinical practice.

## Materials and methods

### Cells and reagents

The HMCLs ARP-1 and U266 were generously provided by Dr. Qing Yi (Center for Hematologic Malignancy, Research Institute, Houston Methodist, Houston, TX, USA), and RPMI-8226 and MM1.S were purchased from the National Collection of Authenticated Cell Cultures (Beijing, China). All cell lines were cultured in RPMI-1640 medium containing 10% fetal bovine serum (FBS, Biological Industries, Israel) at 37 °C under 5% CO_2_ atmosphere.

With the approval of the Ethics Committee of the First Affiliated Hospital of Zhejiang University School of Medicine and informed consents obtained from MM patients, we fetched bone marrow samples for the experimental study only. Primary MM cells were sorted by CD138 microbeads(STEM CELL Technologies, Canada), and then cultured in RPMI-1640 medium containing 20% FBS at 37 °C under 5% CO_2_ atmosphere.

Chidamide and venetoclax were purchased from Med Chem Express (New Jersey, USA).

### Cell viability assay

Cell counting kit-8 (CCK-8) assays were employed to detect cell viability. MM cells (5*10^4^ cells/mL) were seeded in 96-well plates, then treated with designated drugs and cultured for the indicated times at 37 °C under 5% CO_2_ atmosphere. Afterwards, the cells were incubated with 10 µL of CCK8 solution (Med Chem Express, USA) for another 2 h at 37 °C. The absorbance was measured by a microplate reader (Bio-Rad, Model 680) at 450 nm. CompuSyn software (ComboSyn Inc., Paramus, NJ, USA) was used to calculate the combination index (CI) of the two drugs, and CI < 1 indicated a synergistic interaction.

### Flow-cytometric analysis of apoptosis and cell cycle

The HMCLs were treated with the designated drugs and cultured for the indicated times, stained with Annexin V-FITC/ PI (Dojindo, Kumamoto, Japan) and PI staining (Multi Sciences, Lianke Bio, China) according to the manufacturer’s instructions, and then analyzed through FACScan flow cytometer (BD Biosciences) to detect the apoptosis and cell cycle. Flow Jo software (v10, Tree Star, Ashland, United States) and Mod Fit LT software (v3.1, Verity Software House, Inc., Topsham, ME, United States) were employed to evaluate the apoptosis and cell cycle data.

### Alkaline comet assay

Individual cellular DNA damage was detected by the alkaline comet assay (Abcam, ab238544, UK). At least 75 cells were analyzed per sample. The tail moment and the percentage of DNA in the comet tail were recorded to measure the DNA damage. The obtained data were analyzed by CASP software (CASP, Wroclaw, Poland).

### Immunoblotting

The treated cells were collected, washed twice by PBS and lysed with RIPA lysis containing protease and phosphatase inhibitors (Thermo Fisher Scientific) for 30 min on ice. The supernatant was loaded and boiled in water for 10 min. 20 µg/lanes of protein were separated by SDS–polyacrylamide gel electrophoresis (PAGE) and then transferred onto PVDF membranes (Merck Millipore, Darmstadt, Germany). The transblotted membranes were blocked for 2 h using 5% nonfat milk for non-phosphorylated protein or 5% Albumin Bovine V (BSA, Solarbio, China) for phosphorylated protein. The blocked membranes were incubated with corresponding primary antibodies at 4 °C overnight. Then, the membranes were washed three times for 15 min each time with TBST, followed by the appropriate second horseradish peroxidase (HRP)-conjugated antibody for 1 h at room temperature. After washing three times again for 15 min with TBST, the protein bands were detected by the Chemi Doc TM MP Imaging System (Bio-Rad). The primary antibodies, including anti-caspase-3, -BCL2, -BCL-X_L_, -MCL1, -P21, -P27, -cyclin D1, -cyclin-E1, -p-ATM, -p-ATR-, -p-CHK1, -p-CHK2 and -GAPDH were obtained from Cell Signaling Technology (MA, USA). Anti-CDK4, -CDK6, -PARP1, -γH2A.X, -KU80 and -Rad51 antibodies were purchased from Abcam (Cambridge, UK).

### Immunohistochemistry

Tumor tissue was fetched from tumor-bearing NOD/SCID mice, the tumor masses were fixed in 4% paraformaldehyde and then embedded in paraffin before sectioning. Finally, immunohistochemical staining was performed to analyze the cleaved caspase-3, CDK6, *γ*H2AX, BCL-X_L_ and BIM.

### MM xenograft model

After approval by the Animal Care and Use Committees of the First Affiliated Hospital of Zhejiang University School of Medicine, six-week-old NOD-SCID mice were subcutaneously injected with ARP-1(5 × 10^6^) cells into the left or right flanks to establish the MM xenograft model. After 7 days, when a lump formed, the mice were randomly divided into four groups and treated with vehicle, chidamide (15 mg/kg), venetoclax (100 mg/kg) and the combination of chidamide and venetoclax daily for 2 weeks. All drugs were administrated intragastrically. Tumor size was monitored every three days with calipers.

### Gene knockdown

BCL-X_L_ were knocked down by lentiviral transduction using a BCL2L1-specific shRNA transfer vector targeting the residues 5’-GCTCACTCTTCAGTCGGAAAT-3’ (BCL-X_L_-sh1), 5’-GTGGAACTCTATGGGAACAAT-3’ (BCL-X_L_-sh2) and 5’-GGCAGGTATGGAAGGGTTTGT-3’ (BCL-X_L_-sh3); a BCL2L11-specific shRNA transfer vector targeting the residues 5’-TCCCTACAGACAGAGCCACAA-3’ was used to knock down BIM (Shanghai Genechem Co., LTD, China).

### Statistical analysis

All data were analyzed by GraphPad Prism 8 and expressed as means ± standard deviation (SD) of at least three independent experiments. The two-tailed Student’s test was used to determine the statistical differences among experimental groups. *P* values less than 0.05 were considered significant (Additional file 2: Fig. S2).

## Supplementary Information

**Additional file 1: Figure S1**. The expression of BCL2, BCL2L1 and MCL1 in HMCLs. Using qPCR to detect mRNA expression of BCL2, BCL2L1 and MCL1 in HMCLs

**Additional file 2: Figure S2**. Co-treatment with chidamide and ventoclax doesn’t affect the expression of HDAC1, 2, 3 and HDAC 10. (A) HMCLs were exposed to chidamide (1ɥM for U266; 2ɥM for ARP-1 and MM1.S) and/or venetoclax (2ɥM for U266; 4ɥM for ARP-1 and MM1.S) for 48 h. Western blotting was employed to detect the expression of the following cell cycle-related proteins: HDAC1, HDAC2, HDAC3 and HDAC10. (B) Protein levels of HDAC1, HDAC2, HDAC3 and HDAC10 were normalized to those of GAPDH and presented as fold changes relative to vehicle controls. Data are presented as the mean ± SD of at least three independent experiments. (ns P>0.05; ∗P < 0.05; ∗∗P < 0.01; ***P<0.001; ∗∗∗∗P < 0.0001).

## Data Availability

All data generated or analyzed during this study are included in this published article and its supplementary files.

## Abbreviations

AML: Acute myeloid leukemia
CI: Combination index
CDKI: Cyclin-dependent kinase inhibitor
CDK: Cyclin dependent kinase
CLL: Chronic lymphocytic leukemia
DDR: DNA damage response
DSB: DNA double-strand breaks
HDAC: Histone deacetylase
HMCL: Human myeloma cell line
MM: Multiple myeloma
RRMM: Relapse or refractory multiple myeloma
MCL: Mantle cell lymphoma
