# Supplementary figures and images for: Chidamide and venetoclax synergistically exert cytotoxicity on multiple myeloma by upregulating BIM expression
Source: Clin Epigenetics. 2022 Jul 7;14:84. doi: 10.1186/s13148-022-01306-7 (PMC9264603; doi:10.1186/s13148-022-01306-7)

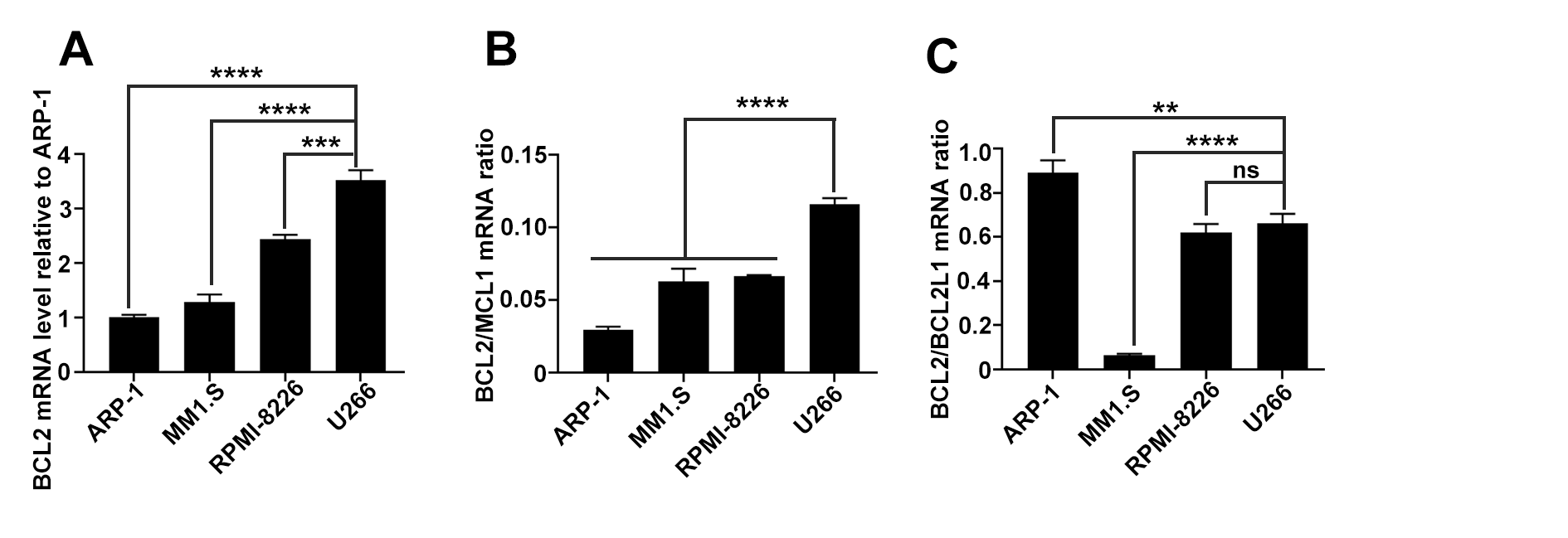

Supplement: Supplementary file 1 — Additional file 1: Figure S1. The expression of BCL2, BCL2L1 and MCL1 in HMCLs. Using qPCR to detect mRNA expression of BCL2, BCL2L1 and MCL1 in HMCLs [file 13148_2022_1306_MOESM1_ESM.tif]

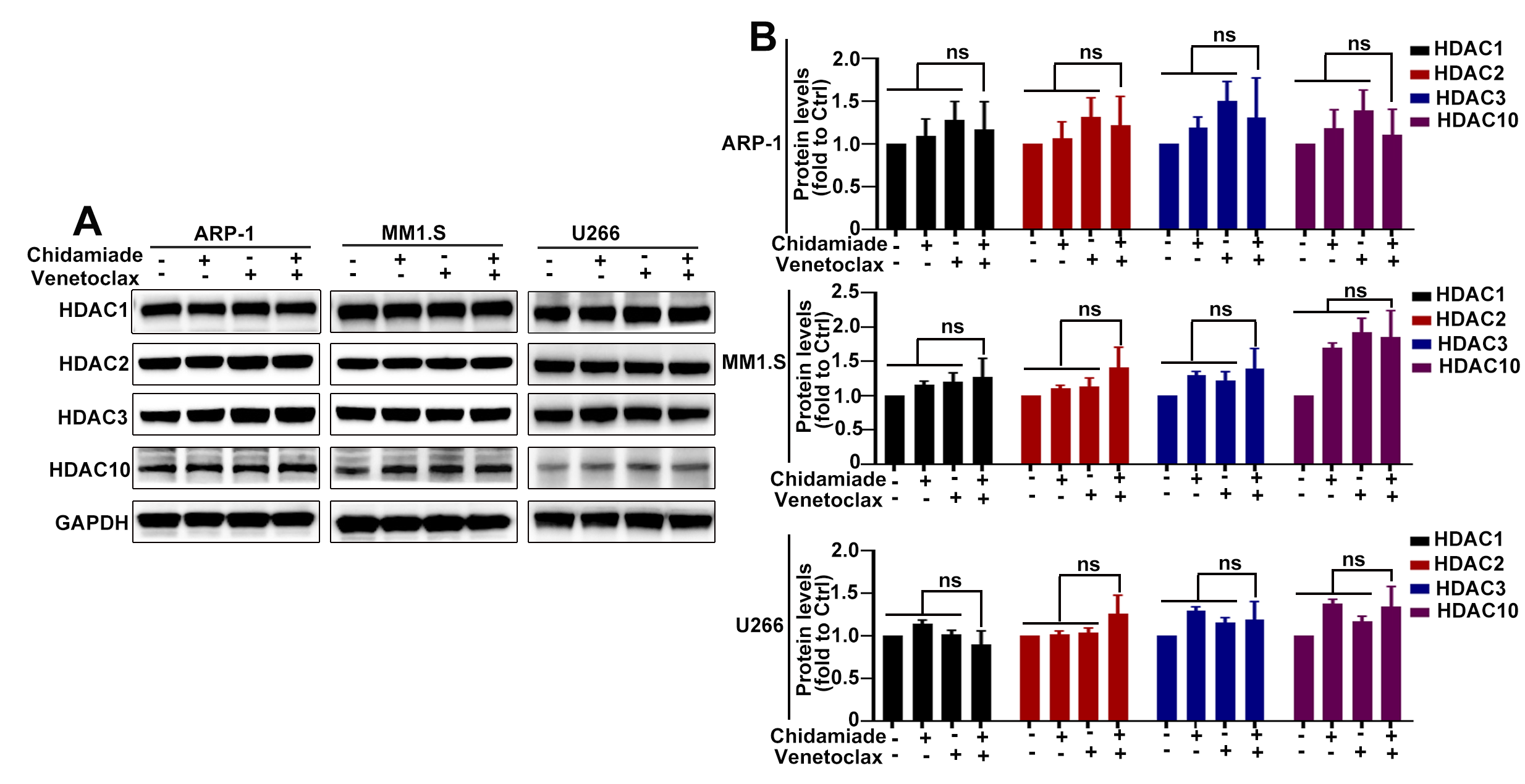

Supplement: Supplementary file 2 — Additional file 2: Figure S2. Co-treatment with chidamide and ventoclax doesn’t affect the expression of HDAC1, 2, 3 and HDAC 10. (A) HMCLs were exposed to chidamide (1ɥM for U266; 2ɥM for ARP-1 and MM1.S) and/or venetoclax (2ɥM for U266; 4ɥM for ARP-1 and MM1.S) for 48 h. Western blotting was employed to detect the expression of the following cell cycle-related proteins: HDAC1, HDAC2, HDAC3 and HDAC10. (B) Protein levels of HDAC1, HDAC2, HDAC3 and HDAC10 were normalized to those of GAPDH and presented as fold changes relative to vehicle controls. Data are presented as the mean ± SD of at least three independent experiments. (ns P>0.05; ∗P < 0.05; ∗∗P < 0.01; ***P<0.001; ∗∗∗∗P < 0.0001). [file 13148_2022_1306_MOESM2_ESM.tif]
